# In Vitro Efficacy of Water-Soluble Mercaptopyridine-Substituted Oxotitanium (IV) Phthalocyanine Compounds in Photodynamic Therapy of Oral Squamous Cell Carcinoma

**DOI:** 10.3390/medicina61071285

**Published:** 2025-07-17

**Authors:** Merve Çiftçi, Mansur Doğan, Didem Duman, Özge Göktuğ Temiz, Mahmut Durmuş, Efkan Bağda

**Affiliations:** 1 Division of Otorhinolaryngology, Tokat Erbaa State Hospital, 60500 Tokat, Türkiye; mervekocciftci@gmail.com; 2 Department of Otorhinolaryngology, Faculty of Medicine, Sivas Cumhuriyet University, 58140 Sivas, Türkiye; 3 Department of Molecular Biology and Genetics, Faculty of Science, Sivas Cumhuriyet University, 58140 Sivas, Türkiye; didemdumann@gmail.com (D.D.); efkanbagda@gmail.com (E.B.); 4 Department of Chemistry, Gebze Technical University, 41400 Gebze, Türkiye; ozgegoktug90@gmail.com; 5 Pharmaceutical Development Department of R&D Center, Abdi Ibrahim Pharmaceuticals, 34538 Istanbul, Türkiye; durmus@gtu.edu.tr

**Keywords:** oxotitanium (IV) phthalocyanine, SCC-9, oral squamous cell carcinoma (OSCC), PDT

## Abstract

*Background and Objectives*: The efficacy of newly synthesized water-soluble octa-mercaptopyridine-substituted oxotitanium (IV) phthalocyanine (oxo-TiPc) and copper (II) phthalocyanine (CuPc) compounds in photodynamic therapy (PDT) was investigated using human tongue squamous cell cancer cell line (SCC-9, ATCC) cultures. *Materials and Methods*: A laser light source with a wavelength of 635 nm was used for this study. The cytotoxic values of the cancerous (SCC-9) and healthy (L-929) cell samples to which different Pc concentrations were applied under laser light were evaluated spectroscopically with the XTT method. *Results*: The oxo-TiPc compound exhibited a significantly lower IC50 value (46.8 µM) for SCC-9 cells compared to the CuPc compound (286.2 µM), indicating higher anticancer activity. This cytotoxicity may be due to decreased aggregation and enhanced reactive oxygen species (ROS) formation. Double-staining tests confirmed that oxo-TiPc-induced cell death included both apoptosis and necrosis. *Conclusions*: The findings show that the oxo-TiPc compound, unlike the CuPc compound, exhibited more selective toxicity to the SCC-9 cell line and has a higher phototoxic effect.

## 1. Introduction

According to GLOBOCAN 2022 estimates, lip and oral cavity cancers are responsible for approximately 2% of cancer diagnoses and deaths, with 389,846 new cases and 188,438 deaths [1]. The Global Cancer Observatory (GCO) predicts that by 2040, the incidence of OSCC will rise by around 40% and the mortality rate will increase accordingly [2]. Squamous cell carcinoma (SCC) accounts for more than 90% of all malignant tumors in the oral cavity. Oral squamous cell carcinoma (OSCC) develops from the mucosal epithelium of the oral region. The asymptomatic, rapid, and aggressive progression and early metastasis of the tumor are responsible for the 5-year survival rate of 39–43%. Clinical diagnosis is usually made in advanced stages, which leads to a high risk of recurrence and a poor prognosis [3]. The primary treatment method includes local tumor removal, chemotherapy, radiotherapy, and their combination. Traditional treatment methods cause tissue and function loss, non-selective toxic effects, the development of drug resistance, limited reuse, and significant esthetic problems. The search for alternative treatment methods that are effective, safe, reproducible, minimally invasive, have few side effects, and do not develop resistance in the treatment of OSCC is increasing every day. Photodynamic therapy (PDT) offers a strong alternative for OSCC treatment with its non-invasive or minimally invasive characteristics, selective toxic effect on cancer cells, no or minimally systemic side effects, not developing drug resistance, suitability for combined use with other treatment methods, and its ability to be used repeatedly. PDT is based on accumulating a non-toxic photosensitizer molecule in the target tissue and its cytotoxic effect by generating reactive oxygen species (ROS) when stimulated by light at the wavelength at which it has maximum absorption. It consists of three basic components, namely (i) a photosensitizer molecule, (ii) light of an appropriate wavelength, and (iii) molecular oxygen [4]. While phthalocyanines absorb at ~635 nm, limiting tissue penetration to superficial tumors (<0.2 cm), their selectivity and photodynamic efficacy make them suitable for early-stage oral squamous cell carcinoma (OSCC). In contrast, chlorin- and bacteriochlorin-based photosensitizers, with an absorption >660 nm, are preferred for deeper tumors (>0.5 cm) [5]. The photosensitizer molecule is of critical importance for effective PDT. Therefore, scientists are working to develop new photosensitizer agents that are non-toxic on their own, have high singlet oxygen yield, can selectively accumulate in the target tissue, and are water-soluble.

Phthalocyanine compounds are very useful vehicles in PDT. These compounds have B and Q absorption bands located in the near-ultraviolet (UV) and near-infrared (IR) regions, respectively. The position of these peaks can be adjusted with appropriate molecular designs as needed. When these compounds are excited, they can convert molecular oxygen into reactive oxygen species, which allows them to be used in many biological applications. The water solubility of these compounds is important for bioapplications [5].

There are studies showing that metal complexes exhibit different anticancer activities. In addition to the many properties of the complexes, metals can modulate the photophysical, photochemical, and binding properties with biomolecules. The magnetic properties of metals are also important in terms of activity in photodynamic therapy. For this reason, a large number of metal complexes have been investigated in terms of cancer studies, and it has been observed that some compounds give quite promising results [6].

In the literature, titanium (Ti) (IV) and Cu(II) metal phthalocyanine compounds have been studied. Copper complexes are promising compounds in cancer treatment, and these complexes have been shown to possess anticancer properties [7]. Copper(II) phthalocyanine compounds are promising in cancer treatment due to their ability to modulate the reduction potential of the Cu(II)/Cu(I) system, facilitating reactive oxygen species (ROS) formation, which enhances anticancer activity through photodynamic mechanisms. The reduction potential of the Cu(II)/Cu(I) system depending on the environment is important in terms of anticancer activity in chemotherapy and photodynamic pathways. Copper induces the formation of reactive oxygen species to fragment DNA. Similarly, the light-assisted excitation of Cu(II) complexes in the red region of the electromagnetic spectrum helps in the generation of different reactive species and thus initiates anticancer activity through a photodynamic mechanism. Overall, in vivo and in vitro studies have shown that the application of copper ions is effective against various cancer cell lines. However, there are studies in the literature showing that the anticancer properties of titanium remain limited [8]. There are also studies showing the use of Ti nanomaterials because of their various advantages [9]. The selection of titanium (Ti) as the central metal for oxo-TiPc was based on its axial oxygen ligand, which reduces aggregation compared to zinc (Zn) or aluminum (Al) phthalocyanines, potentially enhancing water solubility and ROS generation in aqueous environments, despite the higher singlet oxygen quantum yields of ZnPc and AlPc [6]. The compounds containing mercaptopyridine groups have been reported in the literature to exhibit anticancer activity [10]. Hong et al. have shown that organotin (IV) compounds carrying pyridine groups interact with many biomolecules and exhibit antitumor activity through in vitro experiments [11].

This study aims to evaluate the in vitro efficacy of a newly synthesized water-soluble oxotitanium (IV) phthalocyanine (oxo-TiPc) compared to copper (II) phthalocyanine (CuPc) in PDT for oral squamous cell carcinoma (OSCC), contributing new insights into the potential of oxo-TiPc as a selective and effective photosensitizer for OSCC treatment. The objectives of this study are to synthesize and characterize a water-soluble oxotitanium (IV) phthalocyanine (oxo-TiPc), compare its photodynamic efficacy and selectivity against oral squamous cell carcinoma (OSCC) cells with copper (II) phthalocyanine (CuPc), and investigate the mechanisms of cell death induced by these compounds.

## 2. Materials and Methods

**Figure 1:**
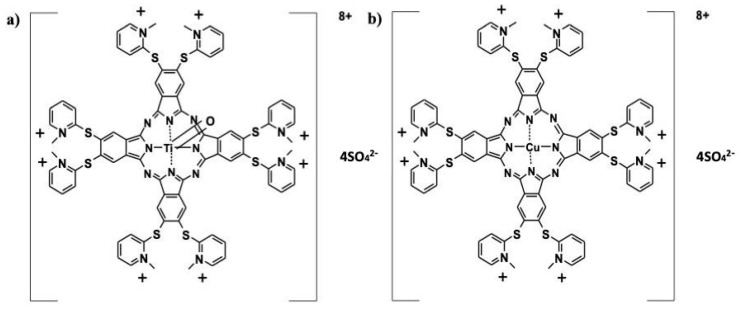
Chemical structures of (**a**) oxotitanium (IV) (oxo-TiPc) and (**b**) copper (II) (CuPc) phthalocyanine compounds. oxo-TiPc: 2,3,9,10,16,17,23,24-Octakis-[(N-methyl-2-mercaptopyridine)phthalocyaninato]oxotitanium (IV) sulfate. CuPc: 2,3,9,10,16,17,23,24-Octakis-[(N-methyl-2-mercaptopyridine)phthalocyaninato]copper (II) sulfate.

2-Mercaptopyridine was purchased from Fluka (AG, Buchs, Switzerland). 4,5-Dichlorophthalonitrile and 4,5-bis (2-mercaptopyridine)phthalonitrile were synthesized and purified according to the procedures described in the literature [12,13]. The water-soluble octa-mercaptopyridine-substituted copper (II) (CuPc) sulfate (Figure 1) phthalocyanine compound used in this study was synthesized and characterized by our group in previously published work [14]. The chemical structures of the studied compounds are shown in Figure 1a (oxo-TiPc) and Figure 1b (CuPc).

### 2.1. Synthesis

2,3,9,10,16,17,23,24-Octakis-[(2-mercaptopyridine)phthalocyaninato]oxotitanium (IV).

4,5-Bis(2-mercaptopyridine) phthalonitrile (1.0 g, 2.9 mmol), urea (0.06 g), and DBU (4–5 drops) were mixed for a while at 70–80 °C under a nitrogen atmosphere. After adding Ti(OBu)_4_ (0.3 mL), the solution mixture was heated under a nitrogen atmosphere to 120 °C and mixed for 5–6 h. After cooling to room temperature, the product was precipitated by dropping it into n-hexane, and it was collected by using filtration. The green solid product was washed successively with hot n-hexane, ethanol, ethyl acetate, and dichloromethane. Yield: 23%, mp > 240 °C. FT-IR: ν_max_, cm^−1^: 3050 (Ar-CH), 1571 (C=C), 1415, 1406, 1369, 1277, 1120, 1067, 1058, 948 (Ti=O), 749. Calcd. for C_72_H_40_N_16_S_8_TiO: 59.66 C, 2.78 H, 15.46 N. Found: 59.79 C, 2.75 H, 15.49 N.

2,3,9,10,16,17,23,24-Octakis-[(N-methyl-2-mercaptopyridine)phthalocyaninato]oxotitanium (IV) sulfate (oxo-TiPc).

2,3,9,10,16,17,23,24-Octakis-[(2-mercaptopyridine)phthalocyaninato]oxotitanium (IV) (0.3 g, 0.154 mmol) in dry DMF (5 mL) was heated under a nitrogen atmosphere to 120 °C, and dimethyl sulfate (DMS) (0.15 mL, 1.54 mmol) was added dropwise. The reaction was stirred at 120 °C for 12 h. After cooling to room temperature, the product was precipitated by dropping it into acetone, and it was collected by using filtration. The green solid product was washed successively with acetone, ethyl acetate, chloroform, n-hexane, carbon tetrachloride, and diethylether. The resulting hygroscopic product was dried over phosphorous pentoxide. Yield: 94%, mp > 240 °C. FT-IR: ν_max_, cm^−1^: 3077–3046 (Ar-CH), 2954 (Aliph-CH), 1567 (C=C), 1490, 1410, 1213, 1076–1058 (S=O), 1003, 745. ^1^H-NMR (D_2_O), (δ: ppm): 2.13–2.63 (m, Aliph-H, 24H), 7.55–9.79 (m, Ar-H, 40H). MALDI-TOF-MS: *m*/*z* calcd. for C_80_H_64_N_16_S_8_TiO·4SO_4_, 1954.11; found [M + H]^8+^ 246.776. UV–Vis (H_2_O, 1 × 10^−5^ M): λ_max_ nm (log ε) 308 (4.72), 638 (4.20), 708 (4.85). (SHIMADZU UV-2101PC UV-VIS Spectrophotometer, Kyoto 604-8511, Japan (wavelength range: 190~900 nm, Double-Beam, Double-Grating, Resolution 0.1 nm)).

### 2.2. Cell Cultures and Pc Compounds

The photocytotoxicity of the phthalocyanine compounds were evaluated using the human oral squamous cell carcinoma (SCC-9, ATCC) and mouse fibroblast cell lines (L-929, ECACC). During the cultivation of cell lines, the phenol red-containing medium was utilized, whereas they were grown in a phenol red-free medium before, during, and after exposure to light.

The SCC-9 cell line was cultured in Dulbecco’s modified Eagle’s medium/nutrient mixture F-12 (DMEM/Ham’s F-12, 1:1) (Biological Industries, Kibutz Beit Haemek, Israel) supplemented with high D-Glucose (4.5 g/L), L-glutamine (2.5 mM) (Pan-Biotech, Aidenbach, Germany), sodium pyruvate (55 mg/mL), sodium bicarbonate (1.2 g/L), HEPES (15 mM), hydrocortisone (0.4 μg/mL), 10% (*v*/*v*) fetal bovine serum (FBS) (Serox, Mannheim, Germany), and penicillin/streptomycin (1%). L-929 cells were cultured in DMEM with high D-glucose (4.5 g/L) containing L-glutamine (4 mM), sodium pyruvate (1 mM), sodium bicarbonate (3.7 g/L), 10% (*v*/*v*) FBS, and penicillin/streptomycin (1%).

Each cell line was cultured in 75 cm^2^ cell culture flasks at 37 °C and 5% CO_2_ in a humidified atmosphere (Nukleon, Istanbul, Türkiye) until reaching approximately 80–90% confluence, at which point they were detached using trypsin/EDTA and subcultured.

Cell viability experiments were performed in clear, flat-bottomed 96-well plates with 10^4^ cells per well in a final volume of 200 µL of phenol red-free medium. Apoptosis experiments with acridine orange/ethidium bromide (AO/EtBr) double staining were performed in 6-well plates with 2.5 × 10^5^ cells per well in a final volume of 1 mL of phenol-free medium. Phthalocyanine compounds were freshly prepared at 1 mM in phenol red-free media suitable for the cultured cell line and were used in the experiments by diluting with the same medium.

### 2.3. Light Treatment Experimental Setup

The studies were conducted with 4 different experimental groups for PDT applications, namely (i) Pc, (ii) light, (iii) light treatment (Pc + light), and (iv) control groups. In the Pc group, only Pc compounds were applied without light, and in the light group, only light was applied to the cell line without Pc. With these two applications, the toxicity of Pc and light alone to the cell lines were determined. In the light treatment group, Pc was applied to the cell lines at concentrations ranging from 500 to 7.8 μM. Then, cell lines were illuminated at 8 J/cm^2^ for 120 s using a semiconductor laser (SUA 635-500, Ankara, Türkiye) at a wavelength of 635 nm with a power density of 66.7 mW/cm^2^. These parameters were chosen based on prior PDT studies to optimize ROS generation while minimizing thermal effects [4]. All studies were performed in three different experiments, each consisting of 3 replicates.

### 2.4. XTT Cell Viability Analysis

Cell viability was checked using trypan blue and counted with a hemocytometer. Cells were seeded in 96-well plates with 104 cells in each well. After incubation for 24 h for cell adhesion and growth, Pc was added to the wells or not added according to the experimental group. Cells were incubated for 24 h to ensure interaction with Pc. After incubation, an 8 J/cm^2^ laser was applied to the cell line and incubated again. Cell viability was determined with an XTT test 24 h after illumination. The medium was removed, and the cells were washed with 100 µL of phosphate-buffered saline (PBS). After PBS washing, 100 µL of medium without antibiotics and phenol red was added to the cells. XTT reagent (sodium 3′-[1-(phenylaminocarbonyl)-3,4-tetrazolium]-bis(4-methoxy6-nitro)benzene sulfonic acid hydrate) was prepared according to the manufacturer’s instructions (Biological Industries, Kibutz Beit Haemek, Israel). Next, 100 µL of reactant was added to 5 mL of dye, mixed well, and then 50 µL of reagent was added to the cell medium. After 6 h of incubation, absorbance values were measured using a microplate reader at 450 and 630 nm wavelengths (AMR 100; ELISA Reader, Jinan, China). The cell viability percentage was calculated by dividing the absorbance in the treatment group by the absorbance in the control group. IC50 values were determined using Prism 9 software (GraphPad, Boston, MA, USA).

### 2.5. Acridine Orange/Ethidium Bromide (AO/EB) Fluorescent Staining

Acridine orange/ethidium bromide (AO/EB) dual fluorescent staining is a useful method to identify apoptosis-related changes in cell nuclear membranes during the apoptosis process [15]. We used the assay to evaluate the apoptosis in the Pc-supplemented, light-treated SCC-9 cell line.

In the cell suspension counted with trypan blue, 1 mL was added to a 6-well plate to make 2.5 × 10^5^ cells/mL. Pc compounds were prepared in a medium without phenol red and added to the adhered cells and incubated for 1 h, and then 8 J/cm^2^ light was applied. After the application, the medium was discarded, and the cells were detached from the background using trypsinization. The cells were collected with centrifugation (1000 rpm) and suspended in 100 µL of PBS, and 2 µL of AO/EB solution (100 µg/mL each) was added. Next, 20 µL of the cell suspension was transferred to a slide. Apoptotic cells were analyzed under a fluorescence microscope (Olympus BX51, Tokyo, Japan) with 40× magnification using a FITC fluorescent filter (excitation = 475 nm, emission: 530 nm). Cells were photographed and analyzed using ImageJ analysis software (İmage J 1.54g, Wayne Rasband and contributors National Institutes of Health, USA) for a qualitative assessment of apoptosis and necrosis based on fluorescence emission and nuclear morphology [16].

### 2.6. Statistical Analysis

The cell viability results were expressed as mean ± standard deviation (SD), and the data were analyzed using a one-way analysis of variance (ANOVA) test followed by a Tukey post hoc test for multiple comparisons. *p* values of less than 0.05 were considered statistically significant (*p* < 0.05).

## 3. Results

**Figure 2:**
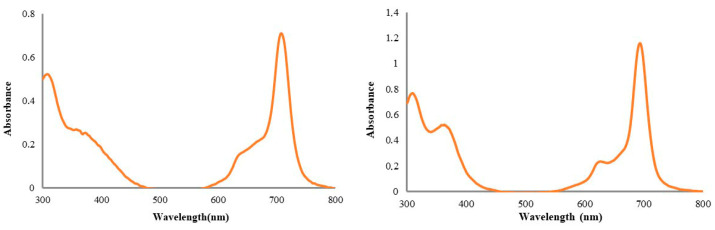
The UV-Vis spectra of newly synthesized water-soluble octa-mercaptopyridine-substituted oxotitanium (IV) phthalocyanine (oxo-TiPc) and copper (II) phthalocyanine (CuPc) compounds in water (1 × 10^−5^ M).

We worked with mercaptopyridine-substituent Pc compounds containing Cu and Ti central atoms for cytotoxicity studies. Generally, the UV-Vis spectra of the phthalocyanine derivatives are consistent with equilibrium between monomeric and dimeric–multimeric forms in an aqueous solution [17]. Aggregation behavior is important for the use of Pc compounds in bioapplications. It has long been thought that the aggregation of phthalocyanines has negative consequences for PDT [18,19]. The studied (oxo-TiPc) and (CuPc) derivatives did not exhibit aggregation in water (Figure 2).

**Figure 3:**
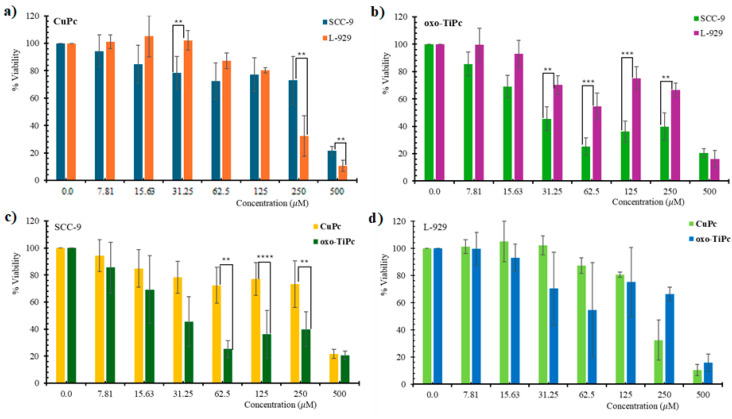
Dose/response curves showing light-stimulated cytotoxic effects of (**a**) oxo-TiPc on SCC-9, (**b**) oxo-TiPc on L-929, (**c**) CuPc on SCC-9, and (**d**) CuPc on L-929 cell lines in the Pc + light and control groups. Pc-only and light-only groups showed >90% viability (see text). Statistical analysis was performed using one-way ANOVA with Tukey’s post hoc test (* *p* < 0.05, ** *p* < 0.01, *** *p* < 0.001, **** *p* < 0.0001).

The cytotoxic effects of oxo-TiPc and CuPc were evaluated across all experimental groups. The Pc-only group (no light) and light-only group (no Pc) showed minimal cytotoxicity, with cell viability remaining above 90% for both SCC-9 and L-929 cell lines at all tested concentrations (*p* > 0.05). In contrast, the Pc + light treatment group demonstrated significant cytotoxicity, with oxo-TiPc exhibiting IC50 values of 44.9 µM for SCC-9 and 198.3 µM for L-929, and CuPc showing IC50 values of 295.5 µM for SCC-9 and 152.9 µM for L-929, indicating a pronounced photodynamic effect (*p* < 0.01)**.** It was determined that the oxo-TiPc compound exhibited higher toxicity to the oral squamous cell carcinoma cell line than the CuPc compound (IC50 = 44.9 and 152.9 µM, respectively, *p* = 0.02) and exhibited more selective toxicity to the SCC-9 cell line than the healthy fibroblast cell line (IC50 = 198.3 and 44.9 µM, respectively, *p* = 0.003) (Figure 3).

This indicates that oxo-TiPc shows high toxicity in the cancerous cell line SCC-9. This effect of CuPc is not as pronounced as it is for oxo-TiPc. This situation contradicts the situation expressed for Ti compounds in the literature [16]. However, it is seen that oxo-TiPc is axially bound to oxygen, which prevented the aggregation of the compound. As is known, phthalocyanines are compounds that are prone to aggregation due to their planar structures and π-π systems, and aggregates reduce the singlet oxygen yield [20]. The decrease in singlet oxygen yield also reduces the effectiveness in photodynamic therapy [21]. The fact that the oxo-TiPc compound showed higher cytotoxicity to cancer cells in our findings may be due to the prevention of aggregation that may increase the ROS under experimental conditions.

**Figure 4:**
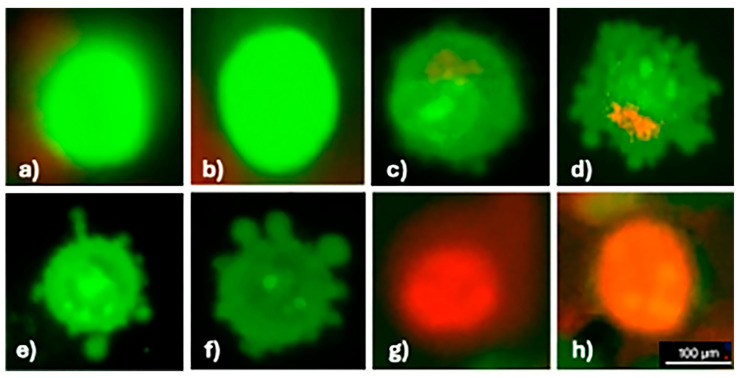
Apoptosis in the SCC-9 cell line detected using AO/EB dual staining. Images show the following: (**a**,**b**) live cells (uniform green fluorescence), (**c**–**f**) apoptotic cells (green/orange fluorescence with chromatin condensation), (**g**,**h**) necrotic cells (uniform red fluorescence). All images were captured at 40× magnification under a fluorescence microscope with a 100 µm scale bar.

Apoptosis in phthalocyanine and light-treated cells was determined morphologically using fluorescence microscopy after AO/EB dual staining. Acridine orange, which penetrates live and dead cells, stains the nucleus with green (dsDNA) and red fluorescence (ssDNA and RNA). Ethidium bromide emits red fluorescence by intercalating into dsDNA in cells with lost membrane integrity. Thus, according to the fluorescence emission and chromatin condensation of the cell nucleus stained with AO/EB double staining, cells are distinguished as (i) live cells with bright green nuclei having a homogeneous structure, (ii) apoptotic cells with heterogeneous and fragmented green, orange, and red nuclei or apoptotic bodies due to chromatin condensation, and (iii) necrotic cells with a homogeneous structure and regular orange or red nuclei [22]. The apoptosis was investigated using the concentrations closest to the IC50 values determined for oxo-TiPc and CuPc compounds, 250 µM and 31.25 µM, respectively. It was found that both necrosis and apoptosis contributed to cell death, and there was no statistically significant difference between the two modes of death (*p* > 0.05) (Figure 4).

## 4. Discussion

Head and neck squamous cell carcinoma (HNSCC) involves the oral cavity, pharynx, nasal cavity, and larynx. Oral cavity cancers are the most common carcinoma in the head and neck, except for non-melanoma skin cancer. Squamous cell carcinoma accounts for 90% of these neoplasms. The tongue, lips, and floor of the mouth are the anatomical subsites where oral cavity tumors are the most common. Tongue carcinoma is classically a disease in older men who have a history of smoking and/or alcohol consumption. In the past, there has been a steady decrease in disease incidence, which can probably be attributed to a general decrease in smoking worldwide. However, studies demonstrate that the incidence of both oral and tongue base squamous cell carcinoma has increased in the last decade, particularly in women and young patients without traditional risk factors such as alcohol and tobacco consumption [23]. This is believed to be related partially to the dramatic increase in HPV-associated oropharyngeal squamous cell carcinoma.

Surgical intervention, which is the primary treatment method, usually has limited efficacy due to the size, stage, and location of the tumor, and often needs to be supported with neoadjuvant/adjuvant applications (additional chemotherapy and radiotherapy). The need to administer high and/or repeated doses of targeted and non-specific chemicals in chemotherapy has significant and often unpredictable side effects. Healthy tissue destruction and lesions that occur after radiotherapy using high-energy rays do not allow for additional surgical interventions in the case of disease recurrence, apart from prolonged recovery periods. The methods employed in cancer treatment are expensive and difficult to apply, in addition to the side effects they cause. Considering all these limitations, it is essential to develop alternative treatment methods to the current treatment methods that have few side effects, high recovery rates, long survival times, and are easy to apply and inexpensive.

PDT represents a new promising cancer treatment method that does not impact the application of other treatment methods in addition to the standard approaches in the medical literature and offers a reproducible alternative treatment if needed. Moreover, clinical studies show that it is highly effective in treating early and recurrent HNSCCs. The use of valid conventional therapies in cancer treatment does not prevent the use of PDT, and PDT does not jeopardize future surgical interventions or radiotherapy [24]. It does not create effects such as increased morbidity or decreased quality of life after surgical intervention or radiation, which have very significant effects.

Upon screening the publications, PDT using first- and second-generation photosensitizing agents was found to be effective in treating severe premalignant dysplasia and early head and neck cancers (Tis, T1, and T2 cancers), with 71–90% complete response rates. Another study was conducted by Karakullukçu et al. [25] with a series of 170 oral and oropharyngeal cancer early-stage (Tis, T1, and T2) cases. They reported an overall response rate of 90.7% and a complete response rate of 70.8% for treatment with temoporfin. The authors indicated that there was a need for further clinical studies, and they expected it to be useful in other cancer treatments.

The difference in incubation times between the XTT viability assay (24 h) and the AO/EB apoptosis assay (1 h) reflects the distinct biological events being evaluated. The longer incubation in the viability assay captures overall cell survival, including delayed cytotoxic effects, whereas the shorter incubation in the apoptosis assay targets early morphological changes associated with apoptosis and necrosis, providing insight into the immediate photodynamic effects of the Pc compounds.

Based on a literature review, it is seen that the photophysical properties of Pc compounds are affected by the presence and nature of the central metal ion. The central metal of phthalocyanines affects their efficiency in PDT. Moghassemi et al. prepared AlPc- and ZnPc-loaded nanoemulsions (AlPc-NE, ZnPc-NE) [26]. According to the results of the 2D cell modeling they performed with ScS, the IC50 value of AlPc-NE was 21.14 ± 0.64 nM, while that of ZnPc-NE was 352.70 ± 20.30 nM. On the other hand, the opposite trend was observed with HL-60 cell lines. In these cells, the IC50 value was found to be 2.78 ± 0.65 for AlPc, while it was 30.15 ± 1.45 nM for ZnPc-NE. Moghassemi et al. found that the NE-encapsulated formulation of ZnPc and AlPc was more effective than the free forms. This was due to several important factors, including the fact that encapsulating the photosensitizers in the nanoemulsion greatly increases their stability and solubility in aqueous environments, which addresses the hydrophobicity of ZnPc and AlPc. Furthermore, because cancers have aberrant vascular architecture, nanoemulsion improves passive targeting through the EPR effect, which enables the nanoparticles to preferentially aggregate in tumor tissues. Using a copper Pc compound, Mir et al. [27] carried out a photodynamic therapy study in 2008 using a photosensitizing agent with two different central atoms, Zn and Cu, on the Jurkat cell line. In their study performed at two different wavelengths by incubating the Jurkat cells with zinc or copper phthalocyanine tetrasulfonate ZnPcS (4) or CuPcS (4), the researchers stated that the zinc-containing compound was more effective than copper.

The most important reason for using TiPc in our present study is that there are few studies on these phthalocyanines in relation to PDT in the literature. However, the titanium phthalocyanines synthesized by Zhang et al. show that the photophysical properties important for photodynamic therapy are quite good [28]. They reported that the Ti–phthalocyanine compounds present high triplet quantum yields (Φ_T_) of 0.81 and 0.85 for TiOPc(β-OPh)_4_ and TiOPc(α-OPh)_4_, while they still maintain a long triplet lifetime (τ_T_) of 80 μs and 69 μs. This makes them produce O_2_(^1^Δg) very efficiently, which is important for possible PDT applications. Our study demonstrates oxo-TiPc’s novel contribution as a water-soluble photosensitizer with high selectivity for SCC-9 cells (IC50 = 44.9 µM) compared to L-929 cells (IC50 = 198.3 µM), outperforming CuPc (IC50 = 295.5 µM for SCC-9).

It is seen that the studies aim to increase the photosensitizing effect of Pc compounds by adding titanium oxide nanoparticles. In the present study, we found cytotoxicity to be statistically significant when titanium was the central atom. Even if the central atom was not titanium, adding it to the compounds increased cytotoxicity. For example, in 2017, Yurt et al. [29] compared ZnPc/ZnPc-integrated TiO_2_ chemicals with the PDT method using the mouse breast cancer (EMT6) and human cervical adenocarcinoma (HeLa) cell lines. The PDT results showed that ZnPc-TiO_2_ could be a potential PDT agent in the treatment of cervical tumors.

The 635 nm absorption of oxo-TiPc limits its clinical application to superficial OSCC tumors (<0.2 cm), unlike far IR-absorbing chlorins or bacteriochlorins effective for thicker tumors (>0.5 cm) [5]. However, its high selectivity and water solubility make it promising for early-stage or recurrent OSCC, though in vivo studies are needed to assess tissue penetration and biodistribution. In vitro assays provide controlled conditions to assess mechanisms such as ROS generation and apoptosis but lack systemic factors including immune responses or vascular effects observed in clinical PDT. Thus, while oxo-TiPc shows promise, further in vivo and clinical studies are essential to validate its therapeutic potential.

This study is limited by its in vitro nature, which lacks the complex tumor microenvironment and systemic factors present in clinical settings, potentially affecting the oxo-TiPc’s efficacy. Another limitation of this study is the use of a single tumor cell line (SCC-9), which may not fully represent the heterogeneity of OSCC. Future studies should evaluate oxo-TiPc’s efficacy in additional OSCC cell lines, such as CAL-27 and HSC-3, to confirm its broader antitumor potential and focus on in vivo models to evaluate tissue penetration and biodistribution, as well as clinical trials to assess therapeutic potential in OSCC patients. Additionally, optimizing photosensitizer delivery systems could further enhance selectivity and efficacy.

## 5. Conclusions

This study demonstrates that the water-soluble oxotitanium (IV) phthalocyanine (oxo-TiPc) exhibits superior photodynamic efficacy against SCC-9 oral squamous cell carcinoma cells (IC50 = 44.9 µM) compared to copper (II) phthalocyanine (CuPc, IC50 = 295.5 µM), with a 6.6-fold lower IC50 and high selectivity over L-929 fibroblast cells (IC50 = 198.3 µM). Compared to the results for zinc phthalocyanine (ZnPc) and aluminum phthalocyanine (AlPc) from prior studies [27], oxo-TiPc’s lower IC50 and reduced aggregation, due to its axial oxygen ligand and mercaptopyridine substitution, suggest enhanced potency and water solubility for photodynamic therapy (PDT). Both apoptosis and necrosis contribute equally to oxo-TiPc-induced cell death (*p* > 0.05), highlighting its versatile cytotoxic mechanism. These findings position oxo-TiPc as a promising photosensitizer for oral squamous cell carcinoma treatment, warranting further in vivo and clinical investigations.

## Data Availability

All data can be requested from the corresponding author.

## Abbreviations

oxo-TiPc	Oxotitanium (IV) phthalocyanine
CuPc	Copper (II) phthalocyanine
PDT	Photodynamic therapy
SCC-9	Human tongue squamous cell cancer cell line
ROS	Reactive oxygen species
GCO	Global Cancer Observatory
SCC	Squamous cell carcinoma
OSCC	Oral squamous cell carcinoma
L-929	Mouse fibroblast cell lines
DMEM	Dulbecco’s modified Eagle’s medium/nutrient mixture F-12
AO/EtBr	Acridine orange/ethidium bromide
HNSCC	Head and neck squamous cell carcinoma
